# Invariant Natural Killer T-cell Dynamics in Human Immunodeficiency Virus–associated Tuberculosis

**DOI:** 10.1093/cid/ciz501

**Published:** 2019-06-12

**Authors:** Naomi F Walker, Charles Opondo, Graeme Meintjes, Nishtha Jhilmeet, Jon S Friedland, Paul T Elkington, Robert J Wilkinson, Katalin A Wilkinson

**Affiliations:** 1 Wellcome Centre for Infectious Diseases Research in Africa, Institute of Infectious Disease and Molecular Medicine, University of Cape Town, Observatory, South Africa; 2 Infectious Diseases and Immunity, and Imperial College Wellcome Trust Centre for Global Health, Imperial College London, United Kingdom; 3 Department of Medicine, University of Cape Town, Observatory, South Africa; 4 Tuberculosis Centre and Department of Clinical Research; 5 Department of Medical Statistics, London School of Hygiene and Tropical Medicine; 6 Institute of Infection and Immunity, St George’s, University of London; 7 National Institute for Health Research Biomedical Research Centre, School of Clinical and Experimental Sciences, Faculty of Medicine, University of Southampton; 8 Francis Crick Institute, London; 9 Department of Medicine, Imperial College London, United Kingdom

**Keywords:** invariant natural killer T cell, HIV, tuberculosis, paradoxical immune reconstitution inflammatory syndrome, innate

## Abstract

**Background:**

Tuberculosis (TB) is the leading cause of mortality and morbidity in people living with human immunodeficiency virus (HIV) infection (PLWH). PLWH with TB disease are at risk of the paradoxical TB-associated immune reconstitution inflammatory syndrome (TB-IRIS) when they commence antiretroviral therapy. However, the pathophysiology is incompletely understood and specific therapy is lacking. We investigated the hypothesis that invariant natural killer T (iNKT) cells contribute to innate immune dysfunction associated with TB-IRIS.

**Methods:**

In a cross-sectional study of 101 PLWH and HIV-uninfected South African patients with active TB and controls, iNKT cells were enumerated using α-galactosylceramide-loaded CD1d tetramers and subsequently functionally characterized by flow cytometry. In a second study of 49 people with HIV type 1 (HIV-1) and active TB commencing antiretroviral therapy, iNKT cells in TB-IRIS patients and non-IRIS controls were compared longitudinally.

**Results:**

Circulating iNKT cells were reduced in HIV-1 infection, most significantly the CD4^+^ subset, which was inversely associated with HIV-1 viral load. iNKT cells in HIV-associated TB had increased surface CD107a expression, indicating cytotoxic degranulation. Relatively increased iNKT cell frequency in patients with HIV-1 infection and active TB was associated with development of TB-IRIS following antiretroviral therapy initiation. iNKT cells in TB-IRIS were CD4^+^CD8^–^ subset depleted and degranulated around the time of TB-IRIS onset.

**Conclusions:**

Reduced iNKT cell CD4^+^ subsets as a result of HIV-1 infection may skew iNKT cell functionality toward cytotoxicity. Increased CD4^–^ cytotoxic iNKT cells may contribute to immunopathology in TB-IRIS.

## Teaser

iNKT cells were depleted in patients with advanced HIV infection, most significantly immunoregulatory CD4+ subsets. In patients with HIV-associated TB who developed TB-IRIS, iNKT cells were elevated with increased degranulation compared to non-IRIS patients, implicating iNKT cells in TB-IRIS immunopathology.

Tuberculosis (TB) causes 1.6 million deaths annually and is the leading cause of death in people living with human immunodeficiency virus type 1 infection (PLWH) [1]. Antiretroviral therapy (ART)–naive PLWH with active TB are at risk of the paradoxical TB immune reconstitution inflammatory syndrome (TB-IRIS) after commencing ART [2]. Paradoxical TB-IRIS is characterized by an acute inflammatory response to *Mycobacterium tuberculosis* presenting as a clinical deterioration in a patient already receiving TB treatment, typically around 2 weeks after ART initiation [3]. Paradoxical TB-IRIS is difficult to manage, frequently requiring nonspecific immunosuppression with corticosteroids. Risk factors include disseminated TB and low CD4 T-cell count at ART initiation, but the pathophysiology is incompletely defined [4]. Recent studies have identified potential contributory innate immune mechanisms, including neutrophil recruitment, inflammasome activation, and proinflammatory cytokine excess [5–10]. These potential mechanisms have been recently reviewed [2].

Invariant natural killer T (iNKT) cells are a T-cell subset that bridge innate and adaptive immunity, and as such are of interest in TB-IRIS pathogenesis [11]. Distinct from natural killer cells and conventional T cells, iNKT cells express an invariant T-cell receptor comprised of Vα24 and Vβ11 in humans, and specifically recognize CD1d-presented lipid antigens, responding on activation with rapid cytokine production. Additionally, iNKT cells recognize and are potently activated by the marine sponge glycolipid α-galactosylceramide (α-galcer), bound to CD1d [12, 13].

*Mycobacterium tuberculosis* cell wall is lipid-rich and therefore CD1d-presented molecules that activate iNKT cells may have a role in host immunity to *M. tuberculosis* [14, 15]. In vitro, iNKT cells directly restricted *M. tuberculosis* growth and were bactericidal [16]. In mice, augmenting iNKT cell responses with α-galcer improved BCG vaccine efficacy and antituberculosis treatment responses [17, 18]. In nonhuman primates, increased iNKT cell frequency was associated with TB resistance [19]. In humans, a limited number of studies have demonstrated numerical and functional defects of iNKT cells in active TB [20–23].

We previously reported elevated expression of cytotoxic mediators, perforin and granzyme B, in peripheral blood mononuclear cells (PBMCs) in response to *M. tuberculosis* antigen stimulation and elevated frequencies of cytotoxic cells expressing CD3 and Vα24 T-cell receptor in TB-IRIS patients compared to non-IRIS controls, suggesting that iNKT cells may play a role in TB-IRIS [24]. Here, we systematically investigated iNKT cells in cross-sectional and longitudinal studies addressing the hypothesis that iNKT cell dysfunction contributes to TB-IRIS immunopathology. We describe for the first time iNKT cell aberration in human immunodeficiency virus (HIV)–associated TB disease and increased cytotoxic iNKT cells in individuals with TB-IRIS.

## METHODS

Full methods are provided in the Supplementary Data.

### Study Participants

Cross-sectional study participants were retrospectively designated into 4 categories: (1) HIV-uninfected participants without active TB (HIV^–^TB^–^); (2) HIV-uninfected participants with a new diagnosis of active TB (HIV^–^TB^+^); (3) ART-naive PLWH without active TB (HIV^+^TB^–^); and (4) ART-naive PLWH with a new diagnosis of active TB (HIV^+^TB^+^).

Longitudinal study participants were ART-naive PLWH with a CD4 count <200 cells/μL and recently diagnosed TB. Longitudinal study visits occurred at TB diagnosis (TB0), ART initiation (ARV0), 2 (ARV2) and 4 (ARV4) weeks of ART and if new symptoms suggesting TB-IRIS occurred. TB-IRIS diagnosis was assigned retrospectively on expert case review, using consensus criteria [3].

The study was approved by the University of Cape Town Human Research Ethics Committee (reference number 516/2011). All participants provided written informed consent.

### iNKT Cell Enumeration and Characterization

PBMCs were isolated over Ficoll and cryopreserved. Cells were rapidly thawed in warmed Roswell Park Memorial Institute medium/10% fetal calf serum (FCS), before viability staining with Violet LIVE/DEAD Fixable stain kit (VIVID, Invitrogen, Paisley, United Kingdom), then washed and resuspended for incubation with either α-galcer–loaded CD1d tetramer or control CD1d tetramer (Proimmune, Oxford, United Kingdom) for 30 minutes on ice, protected from light. Subsequently, cells were washed, stained with antibody mastermix 1 (Supplementary Table 1) for 30 minutes at 4°C, washed and resuspended in phosphate-buffered saline, 1% Hi-FCS, and 2% paraformaldehyde, then incubated for 1 hour, washed, and resuspended for acquisition.

### Data Acquisition and Analysis

**Figure 1.**
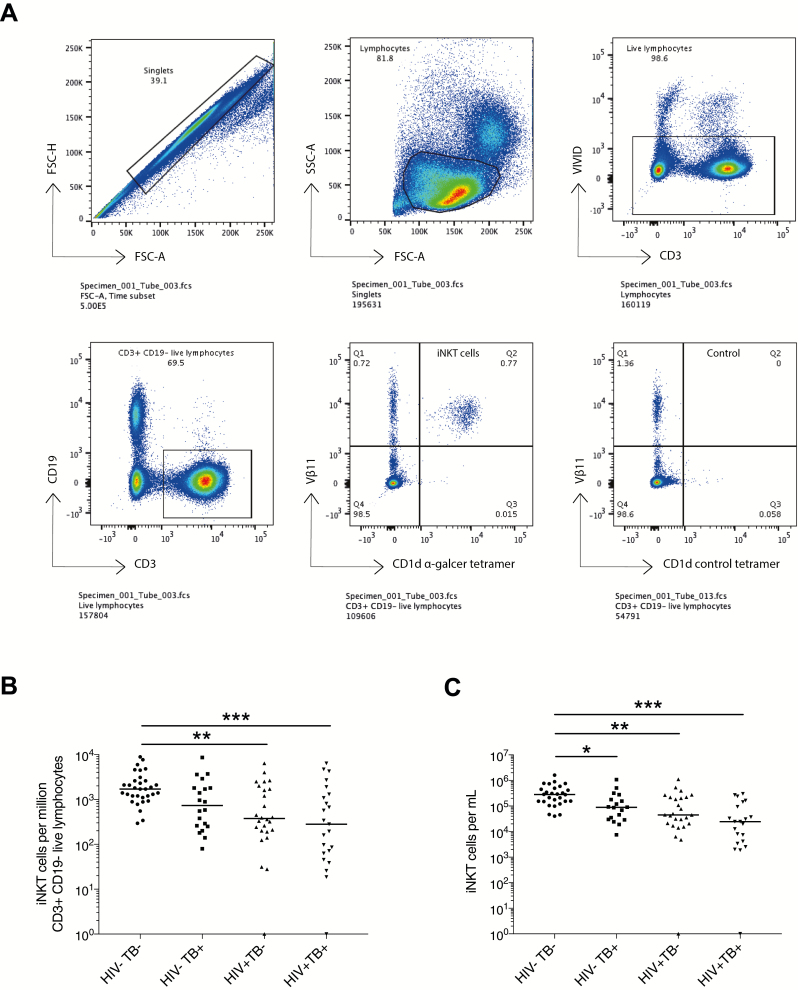
Reduced invariant natural killer T (iNKT) cells in human immunodeficiency virus type 1 (HIV-1) infection and active tuberculosis (TB). iNKT cells were enumerated by flow cytometry using ⍺-galcer–loaded CD1d tetramers. Each sample was stained in parallel with a control tetramer (without ⍺-galcer) to identify nonspecific tetramer binding for subtraction. *A*, The gating strategy demonstrates an iNKT cell frequency of 0.77%, with no control tetramer binding, equivalent to 7700 cells per million CD3^+^CD19^–^ live lymphocytes. *B*, Decreased iNKT cell frequency was found in people living with HIV (PLWH) with active TB (HIV^+^TB^+^) and without active TB (HIV^+^TB^–^), compared to HIV-uninfected patients without active TB (HIV^–^TB^–^). Similarly, in PLWH with and without active TB, decreased iNKT cell numbers (cells/mL peripheral blood) (*C*) were found compared to HIV^–^TB^–^ patients. Additionally, in HIV-uninfected patients with active TB (HIV^–^TB^+^), iNKT cell numbers were reduced compared to HIV^–^TB^–^ patients. Analysis was by Kruskal-Wallis test with Dunn multiple comparisons test to calculate adjusted *P* values: *P < .05; **P < .01; ***P < .001. In (*B*) and (*C*), zero values were replaced by 1 for representation on a log scale. Abbreviations: α-galcer, α-galactosylceramide; FSC-A, forward scatter area; FSC-H, foward scatter height; SSC-A, side scatter area; VIVID, Violet LIVE/DEAD Fixable stain.

Data were acquired on an LSRFortessa (BD Biosciences) and analyzed using FlowJo software (Tree Star, Ashland, Oregon). iNKT cells were defined as CD3^+^ CD19^–^CD1d α-galcer tet^+^ Vβ11^+^ T cells. The gating strategy is shown in Figure 1A. iNKT cell frequency was calculated as a percentage of CD3^+^CD19^–^ live lymphocytes, with subtraction of the equivalent tetramer negative control proportion, and reported per million CD3^+^CD19^–^ live lymphocytes. iNKT cell numbers were calculated by multiplying the iNKT cell frequency as a percentage of live lymphocytes with the total lymphocyte count per milliliter of peripheral blood [22].

Statistical analysis was performed using Prism 6 (GraphPad) and Stata 14 software. Unadjusted nonparametric analyses were by 2-tailed Fisher exact or Mann-Whitney *U* test, or for comparisons of >2 groups, by Kruskal-Wallis test with Dunn multiple comparisons test. In the cross-sectional study, we used a multivariable linear regression model to investigate differences in iNKT cell frequency and in percentage iNKT cell CD4/CD8 expression by disease category. In the longitudinal study, a multivariable negative binomial model was fitted to examine associations of iNKT cell frequency and number with TB-IRIS status, and a multivariate linear regression model to estimate difference in CD4/CD8 cell subset percentages between TB IRIS and non-IRIS patients.

## RESULTS

PBMC samples were available from 101 patients (Table 1). HIV^+^TB^+^ patients had lower total CD4 counts but similar CD4 percentages compared with HIV^+^TB^–^ patients, and higher HIV-1 viral loads. In the HIV^+^TB^+^ compared to the HIV-TB^+^ group, there were trends toward reduced cavitary (*P* = .067), but increased miliary and extrapulmonary (*P* = .051 and *P* = .054, respectively) TB presentation, indicating reduced destructive pulmonary pathology but more widely disseminated TB disease in PLWH [25].

**Table 1.** Demographic and Clinical Features of the Cross-sectional Study Participants

Characteristic	HIV^–^TB^–^	HIV^–^TB^+^	HIV^+^TB^–^	HIV^+^TB^+^	*P* Value
Total No.	32	20	26	23	
Female sex	14 (43.8)	7 (35.0)	15 (57.7)	9 (39.1)	>.100^a^
Current or former smoker	17 (53.1)	10 (50.0)	9 (34.6)	9 (39.1)	>.100^a^
Age, y, median (IQR)	29.0 (23.3–38.8)	38.0 (30.0–42.8)	32.5 (28.5–35.3)	31.0 (28.0–40.0)	.059^b^
CD4 T-cell count, median cells/mL (IQR)	NA	NA	349 (204–483)	187 (104–386)	**.041**
CD4 T-cell %, median (IQR)	NA	NA	17.8 (12.0–22.3)	13.7 (9.22–26.3)	.901
HIV viral load, median copies/mL (IQR)	NA	NA	25 735 (6807–92 169)	296 196 (13 540–503 097)	**.031**
Symptomatic	16 (50.0)	20 (100)	10 (38.5)	23 (100)	
Duration of symptoms, d, median (IQR)	14.0 (4.00–150)	28.0 (14.0–30.0)	60.0 (11.3–82.5)	30.0 (21.0–30.5)	.595^c^
Miliary TB	0 (0)	0 (0)	0 (0)	5 (21.7)	.051^d^
Extrapulmonary TB	NA	3 (15.0)	NA	10 (43.5)	.054^d^
Smear-positive TB	0 (0)	13 (65.0)	0 (0)	8 (34.8)	.069^d^
Culture-positive TB	0 (0)	10 (50.0)	(0)	18 (78.3)	.064^d^
Clinical diagnosis of TB	0 (0)	3 (15.0)	0 (0)	2 (8.70)	.650
Cavitary disease on CXR	0 (0)	14 (70.0)	0 (0)	9 (39.1)	.067^d^

Data are presented as no. (%) unless otherwise indicated. *P* values <.05 are highlighted in bold.

Abbreviations: +, infected; –, uninfected; CXR, chest radiograph; HIV, human immunodeficiency virus; IQR, interquartile range; NA, not applicable; TB, tuberculosis.

^a^For comparison between each group by Fisher exact test.

^b^For comparison of all groups by Kruskal-Wallis, Dunn multiple comparisons test for a difference between HIV^–^TB^–^ and HIV^–^TB^+^ (*P* = .033).

^c^For comparison of all groups by Kruskal-Wallis test.

^d^For comparison between HIV^–^TB^+^ and HIV^+^TB^+^ by Fisher exact test.

### Circulating iNKT Cells Are Depleted in HIV-1 Infection and Active TB

In an unadjusted analysis, comparing iNKT cell frequency in PLWH and HIV-1–uninfected patients, with and without active TB, we found that iNKT cell frequency was reduced in HIV^+^TB^+^ (*P* = .001) and HIV^+^TB^–^ (*P* = .005) compared with HIV^–^TB^–^ (Figure 1B and Table 2). Example plots are shown in Supplementary Figure 1. A similar pattern was observed in comparison of iNKT cell numbers (iNKT cells/mL; Figure 1C), and reduction in iNKT cell numbers was found in HIV^–^TB^+^ compared with HIV^–^TB^–^ patients (*P* = .044). Linear regression comparing HIV^–^TB^+^, HIV^+^TB^–^, and HIV^+^TB^+^ to HIV^–^TB^–^ provided further evidence of association between reduced iNKT cell frequency in HIV^+^TB^–^ (*P* = .023) and HIV^+^TB^+^ (*P* = .024) after adjustment for age and sex, but there was no evidence of a reduction in iNKT cell frequency in HIV^–^TB^+^ compared to HIV^–^TB^–^ (*P* = .301).

**Table 2.** Invariant Natural Killer T Cell Enumeration in Cross-sectional Study Participants, by Diagnosis

Patient Category				Dunn Multiple Comparisons Test				
HIV^–^TB^–^	HIV^–^TB^+^	HIV^+^TB^–^	HIV^+^TB^+^	HIV^–^TB^–^ vs HIV^–^TB^+^	HIV^–^TB^–^ vs HIV^+^TB^–^	HIV^–^TB^–^ vs HIV^+^TB^+^	HIV^+^TB^–^ vs HIV^+^TB^+^	HIV^–^TB^+^ vs HIV^+^TB^+^
iNKT cell frequency, median per million CD3^+^CD19^–^ live lymphocytes (IQR)								
1700 (1125–2600)	735 (253–1800)	375 (198–1775)	280 (62.7–1300)	.149	**.005**	**.001**	>.999	.731
iNKT cell number, median cells/mL blood (IQR)								
282 628 (151 100–487 870)	88 580 (29 600–203 771)	44 965 (19 635–219 669)	24 439 (3789–119 449)	**.044**	**.002**	**<.001**	.432	.161
CD4^+^ iNKT cells, median percentage (IQR)								
44.5 (27.9–61.1)	38.9 (16.3–67.1)	13.67 (2.77–39.2)	3.15 (0–39.6)	>.999	**.005**	**<.001**	>.999	**.007**
CD4^+^ iNKT cell frequency, median per million CD3^+^CD19^–^ live lymphocytes (IQR)								
712 (451–1043)	202 (78.8–601)	100 (11.5–236)	18.9 (0–148)	**.016**	**<.001**	**<.001**	>.999	**.013**

Abbreviations: HIV, human immunodeficiency virus; iNKT, invariant natural killer T; IQR, interquartile range; TB, tuberculosis. *P* values <.05 are highlighted in bold.

### CD4^+^ iNKT Cell Subsets Are Depleted in HIV-1 Infection

**Figure 2.**
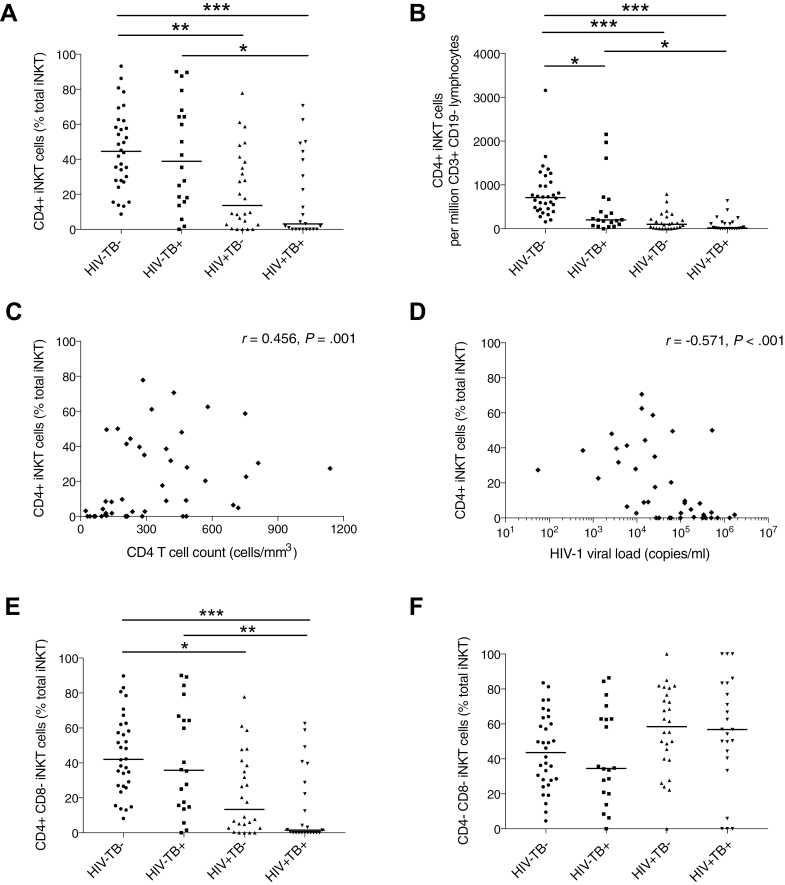
CD4^+^ invariant natural killer T (iNKT) cell subset depletion in human immunodeficiency virus type 1 (HIV-1)–associated tuberculosis (TB). People living with HIV (PLWH), most significantly those with active TB, had depleted CD4^+^ iNKT cells as measured by percentage of total iNKT cell count (*A*) and frequency per million CD3^+^CD19^–^ live lymphocytes (*B*). In PLWH, peripheral blood CD4 T-cell count positively correlated with CD4^+^ iNKT cell percentage (*C*). HIV-1 viral load negatively correlated with CD4^+^ iNKT cell percentage (*D*). In HIV-uninfected patients without active TB, iNKT cells were mostly either CD4^+^CD8^–^ or double negative (CD4^–^CD8^–^), whereas in PLWH, CD4^+^CD8^–^ iNKT cells were depleted and double-negative iNKT cells were the predominant subset (*E* and *F*). Analysis was by Kruskal-Wallis test with Dunn multiple comparisons test to calculate multiplicity-adjusted *P* values: *P < .05; **P < .01; ***P < .001 or by Spearman correlation (*C* and *D*).

iNKT cells may exist as CD4^+^CD8^–^, CD4^–^CD8^+^, CD8^+^CD4^+^, or double-negative (DN) subsets. CD4^+^ iNKT cells secrete both Th1 and Th2 cytokines and may be immunoregulatory, whereas CD8^+^ iNKT cells and DN iNKT cell subsets predominantly secrete Th1 cytokines and have increased cytotoxic functionality [26, 27]. Unadjusted analyses showed that HIV-1 infection was associated with lower CD4^+^ iNKT cell percentages (Figure 2A) and frequency (CD4^+^ iNKT cells per million CD3^+^CD19^–^ live lymphocytes; Figure 2B) in patients with (*P* = .007) and without (*P* = .005) active TB. Active TB did not clearly reduce CD4^+^ iNKT cell percentage, but was associated with reduced CD4^+^ iNKT cell frequency in HIV-uninfected patients (*P* = .016; Figure 2B). In PLWH, total iNKT cell frequency did not correlate with peripheral blood CD4 T-cell count, peripheral blood CD4 T-cell percentage, or HIV-1 viral load. However, CD4^+^ iNKT cell percentage was correlated with total peripheral blood CD4 T-cell count (*r* = 0.456, *P* = .001; Figure 2C) and there was an inverse correlation with HIV-1 viral load (*r* = –0.571, *P* < .001; Figure 2D), indicating that most severe depletion of CD4^+^ iNKT cells occurred in advanced HIV infection.

Next, we examined CD4 and CD8 coexpression on iNKT cells. In HIV^–^TB^–^, we found CD4^+^CD8^–^ and DN iNKT cells (Figure 2E and 2F, respectively) to be the predominant iNKT cell subsets constituting a median of 42.1% and 43.7% of the iNKT cell population, respectively. However, compared to HIV^–^TB^–^ patients, PLWH had reduced percentages of CD4^+^CD8^–^ iNKT cells, constituting a median of only 1.55% iNKT cells in HIV^+^TB^+^ (*P* < .001). In PLWH, there was a trend toward an increased percentage of CD4^–^CD8^–^ iNKT cells (Figure 2F) and CD4^–^CD8^+^ iNKT cells (not shown), compared to HIV-uninfected patients. To explore this further, we performed regression analysis comparing CD4/CD8 iNKT cell percentages in each group to HIV^–^TB^–^, adjusting for age and sex (Supplementary Table 2). This analysis showed evidence of reduced CD4^+^CD8^–^ iNKT cells in HIV^+^TB^–^ and HIV^+^TB^+^ compared with HIV^–^TB^–^ (*P* < .001 for both) and increased CD4^–^CD8^–^ percentage in HIV^+^TB^–^ (*P* = .010). CD4^–^CD8^+^ iNKT cells were increased in HIV^+^TB^–^ (*P* = .037) and HIV^+^TB^+^ (*P* = .016) compared to HIV^–^TB^–^. For CD4/CD8 subset iNKT cell frequencies, see Supplementary Figure 2.

### iNKT Cells in HIV-associated TB Are Proinflammatory With a Cytotoxic Phenotype

**Figure 3.**
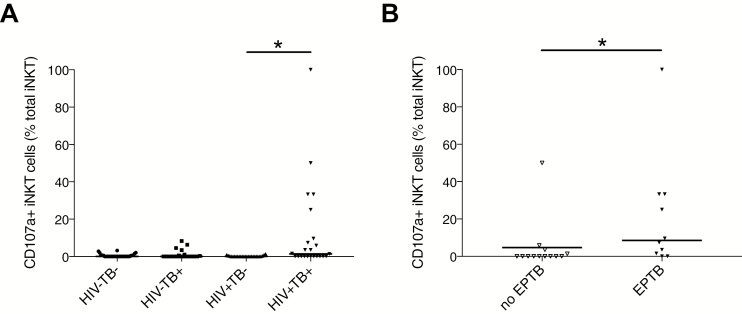
Invariant natural killer T (iNKT) cell cytotoxicity in human immunodeficiency virus type 1 (HIV-1)–associated tuberculosis (TB). In HIV-associated TB, there were increased percentages of CD107a^+^ iNKT cells, suggestive of cytotoxic degranulation (*A*). In people living with HIV (PLWH) with clinical features of extrapulmonary TB (EPTB), there were increased CD107a^+^ iNKT cell percentages compared to PLWH with pulmonary TB (*B*). Analysis was by Kruskal-Wallis test with Dunn multiple comparisons test to calculate multiplicity-adjusted *P* values or by Mann-Whitney *U* test in (*B*): * *P* < .05.

There was high iNKT cell surface expression of the maturation marker, CD161, in addition to CD95 and PD1 in the HIV^+^TB^+^ group, but not more than in the control groups (data not shown). We investigated iNKT cell degranulation by measuring CD107a surface expression [28]. CD107a^+^ iNKT cells were increased in the HIV^+^TB^+^ group, compared with the HIV^+^TB^–^ group, suggesting increased cytotoxic degranulation (Figure 3A), but this phenotype was not observed in all HIV^+^TB^+^ patients. To explore this further, we investigated association between CD107a^+^ iNKT cell positivity and TB disease phenotype in HIV^+^TB^+^. We found significantly increased CD107a^+^ iNKT cell percentage in HIV^+^TB^+^ patients with clinical features of extrapulmonary TB compared to those without, consistent with the hypothesis that disseminated *M. tuberculosis* might lead to peripheral blood iNKT cell degranulation (Figure 3B).

In summary, we found that HIV-1 infection was associated with iNKT cell depletion and CD4^+^ iNKT cell subsets were most significantly depleted in advanced HIV. Active TB was associated with a modest reduction in iNKT cell number in HIV-uninfected patients but did not clearly reduce iNKT cell frequency. The immunoregulatory CD4^+^CD8^–^ iNKT cell subset, the predominant subset in the healthy repertoire, was depleted in PLWH with and without active TB. CD4^–^CD8^+^ and DN iNKT cells were the dominant iNKT cell subsets in PLWH. There were increased CD107a^+^ iNKT cell percentages in PLWH with active TB, indicating a cytotoxic phenotype, which was associated with extrapulmonary TB.

### iNKT Cell Frequency Is Increased in TB-IRIS Patients

Next, in a longitudinal study, we evaluated iNKT cells in patients with advanced HIV and recently diagnosed TB, who commenced TB treatment and then ART, and were at risk of paradoxical TB-IRIS. Fifty-seven participants were enrolled. Clinical features of this cohort have previously been reported [25]. Paradoxical TB-IRIS was diagnosed in 29 (59.2%) patients. Participants were included if PBMCs were available at least at 1 study timepoint (TB0, ARV0, ARV2, and ARV4) and there was follow-up to ARV12. One participant was excluded as no PBMC samples were available, another as they were an elite controller and therefore likely to be immunologically distinct, and a third due to hepatotoxicity on TB treatment, resulting in a significant delay to ART initiation. The subsequent analysis reports findings from 29 TB-IRIS patients and 17 non-IRIS controls. Patient demographics and TB diagnosis are provided in Table 3 and were not significantly different comparing patients in the TB-IRIS group with non-IRIS. Between ARV0 and ARV4, peripheral blood CD4 T-cell counts increased (*P* < .001) from median 101 cells/μL to 206 cells/μL in TB-IRIS patients and from 99 cells/μL to 175 cells/μL in non-IRIS patients.

**Table 3.** Demographic and Clinical Features of Participants in Longitudinal Study at Enrollment

Characteristic	TB-IRIS	Non-IRIS	*P* Value
Total No. (%)	29 (63.0)	17 (37.0)	
Female sex	14 (48.3)	10 (58.8)	.552
Current or former smoker	9 (31.0)	3 (17.5)	.489
Age, y, median (IQR)	35.0 (29.5–42.0)	35.0 (30.5–43.0)	.924
CD4 T-cell count, cells/μL, median (IQR)	89.0 (64.0–141.5)	82.0 (69.5–145.5)	.987
HIV-1 viral load, copies/mL, copies (IQR)	621 075 (207 018–1 185 455)	520 295 (126 925–1 029 554)	.343
Extrapulmonary TB	21 (72.4)	12 (70.6)	1.00
Miliary TB	5 (17.2)	1 (5.88)	.390
Smear-positive TB	14 (48.3)	5 (29.4)	.235
Culture-positive TB	21 (72.4)	11 (64.7)	.742
Clinical diagnosis of TB	2 (6.90)	4 (23.5)	.174
ART initiation, days post–TB treatment initiation, median (IQR)	15 (14–28)	21 (14–41)	.186
IRIS symptom onset, days post–ART initiation, median (IQR)	6 (4–10)	NA	
IRIS presentation, days post–ART initiation, median (IQR)	14 (9–15)	NA	
INSHI criteria for paradoxical TB-IRIS fulfilled	25 (86.2)	0 (0)	

Data are presented as no. (%) unless otherwise indicated.

Abbreviations: ART, antiretroviral therapy; HIV-1, human immunodeficiency virus type 1; INSHI, International Network for the Study of HIV-associated IRIS; IQR, interquartile range; IRIS, immune reconstitution inflammatory syndrome; NA, not applicable; TB, tuberculosis.

**Figure 4.**
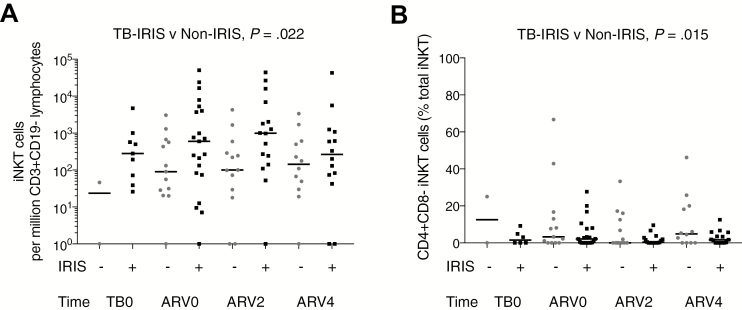
Invariant natural killer T (iNKT) cells are elevated in patients with tuberculosis-associated immune reconstitution inflammatory syndrome (TB-IRIS) and are CD4^+^CD8^–^ subset depleted. iNKT cells were enumerated longitudinally by flow cytometry using ⍺-galcer–loaded CD1d tetramers, in a cohort of 46 patients with active tuberculosis (TB) and human immunodeficiency virus type 1 (HIV-1) infection. Samples were collected around the time of TB diagnosis (TB0), at antiretroviral therapy initiation (ARV0, a median of 17.5 days after TB treatment initiation), and at 2 weeks (ARV2) and 4 weeks (ARV4) post–ART initiation; not all patients contributed data to the first timepoint as patients who had taken >4 doses of TB treatment at enrollment contributed data from ARV0, resulting in fewer data points at TB0. TB-IRIS presentation was typically at ARV2. Increased iNKT cell frequency (*A*) was observed in TB-IRIS patients compared to non-IRIS controls. CD4^+^CD8^–^ iNKT cell percentages were reduced in TB-IRIS patients (*B*). Statistical analysis was by multivariable negative binomial modeling to examine associations of iNKT cell frequency and number with TB-IRIS status and by multivariate linear regression modeling to estimate difference in CD4/CD8 cell subset percentages between TB-IRIS and non-IRIS patients, including data from all timepoints to derive *P* values. In (*A*), zero values were replaced by 1 for representation on a log scale.

First, we enumerated iNKT cells. We found an elevated iNKT cell frequency in TB-IRIS compared to non-IRIS patients (Figure 4A). At ARV2, the most frequent time of TB-IRIS presentation, the median iNKT cell frequency per million CD3^+^CD19^–^ live lymphocytes in TB-IRIS was 992 (interquartile range [IQR], 166–5682) compared to 100 (IQR, 24.5–440) in non-IRIS patients (*P* = .025 in unadjusted analysis). Multivariable modeling including data from timepoints ARV0, ARV2, and ARV4 demonstrated a significant association between TB-IRIS and increased iNKT cell frequency, adjusted for age and sex (*P* = .022; Supplementary Table 3) but no increase in iNKT cell frequency over time, and the association did not differ with total peripheral blood CD4 T-cell count, nor HIV viral load. A similar trend was found for iNKT cell numbers in the adjusted logistic regression analysis (*P* = .062; Supplementary Figure 3).

### iNKT Cell Function and Phenotype in TB-IRIS

Next, we examined CD4/CD8 iNKT cell subsets in the longitudinal study. CD4^+^ iNKT cell percentage and frequency were low, both in TB-IRIS and non-IRIS patients, and did not increase in the first 4 weeks of ART, despite an increased peripheral blood CD4 T-cell count. CD4^+^CD8^–^ iNKT cell percentage was significantly lower in TB-IRIS patients than non-IRIS patients (*P* = .015 by multivariate linear regression modeling; Figure 4B and Supplementary Table 4). Supplementary Figure 4 shows CD4/CD8 subset frequency demonstrating a predominance of DN and CD4^–^CD8^+^ iNKT cells in TB-IRIS compared with non-IRIS patients, at ARV2 (*P* = .029 and *P* = .036, respectively).

**Figure 5.**
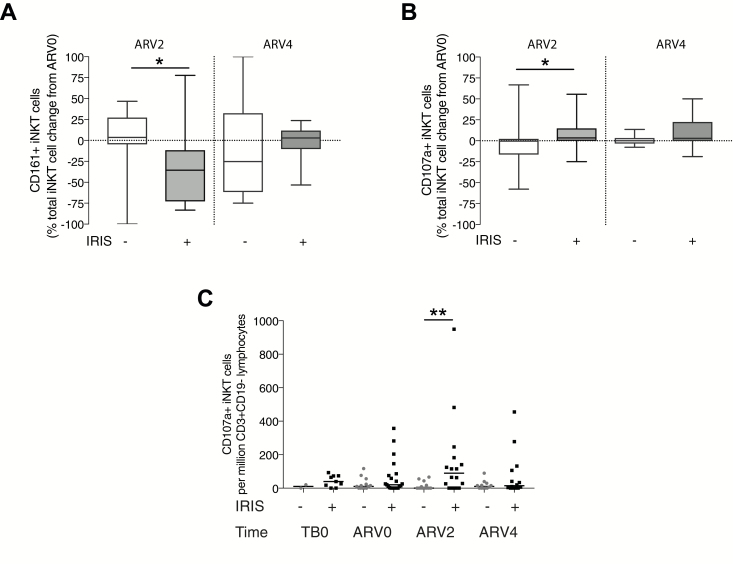
Invariant natural killer T (iNKT) cell cytotoxicity associated with tuberculosis-associated immune reconstitution inflammatory syndrome (TB-IRIS). iNKT cells were characterized longitudinally by flow cytometric analysis for surface markers CD161 and CD107a, in patients with TB-IRIS and without IRIS. Between antiretroviral therapy initiation (ARV0) and 2 weeks post–ART initiation (ARV2), there was a reduction in CD161^+^ iNKT cell percentage in TB-IRIS compared with non-IRIS patients (*A*), whereas CD107a^+^ iNKT cell percentage increased in TB-IRIS patients between ARV0 and ARV2, compared to non-IRIS patients (*B*). CD107a^+^ iNKT cell frequency (cells per million CD3^+^CD19^–^ live lymphocytes) was increased in TB-IRIS patients compared to non-IRIS controls at ARV2, the most common time of TB-IRIS presentation (*C*). Mann-Whitney *U* test for TB-IRIS vs non-IRIS: *P < .05; **P < .01.

In both TB-IRIS and non-IRIS patients, CD161^+^ iNKT cell and CD107a^+^ iNKT cell percentages were dynamic (Supplementary Figure 5*A* and 5*B*). CD95 cell surface expression, indicative of cytotoxicity, and PD1^+^ iNKT cell percentages were high both in TB-IRIS and non-IRIS patients, whereas CD40L^+^ iNKT cell percentages were relatively low, possibly indicating iNKT cell exhaustion (Supplementary Figure 5*C–E*) [22, 29]. In TB-IRIS, CD161^+^ iNKT cell percentages decreased between ARV0 and ARV2, suggesting a loss of mature iNKT cells, whereas in non-IRIS patients, iNKT cell CD161 positivity was similar (Figure 5A). In TB-IRIS patients, CD107a^+^ iNKT cells increased between ARV0 and ARV2 relative to non-IRIS patients, suggesting that degranulation occurred at the time of IRIS symptom onset (Figure 5B). CD107a^+^ iNKT cell frequency was increased in TB-IRIS compared with non-IRIS patients at ARV2 (Figure 5C).

In summary, patients with advanced HIV and active TB had low circulating iNKT cell frequency pre–ART initiation, but iNKT cell populations were skewed toward proinflammatory, cytotoxic subsets. Higher iNKT cell frequency was associated with TB-IRIS following ART initiation, and iNKT cells in TB-IRIS patients were CD4^+^CD8^–^ subset depleted, with increased DN and CD4^–^CD8^+^ iNKT cell frequency at the time of TB-IRIS onset. Increased CD107a^+^ iNKT cell subsets in TB-IRIS patients also at ARV2 suggested increased iNKT cell degranulation occurring at the time of TB-IRIS presentation.

## DISCUSSION

In this study, we demonstrated low iNKT cell frequency in ART-naive patients with advanced HIV infection, with a paucity of CD4^+^ iNKT cells, and relatively increased proportions of CD4^–^CD8^–^ iNKT cells, representing a shift from CD4^+^ subsets found in HIV-uninfected patients. Decreased iNKT cell numbers and CD4^+^ iNKT cell frequency were associated with active TB in patients without HIV infection, but this finding was not consistent in PLWH. In PLWH with active TB, increased degranulation of iNKT cells was found. Despite low iNKT cell frequencies in these patients, there were relatively increased iNKT cells in patients who went on to develop TB-IRIS compared to those who did not, and these were predominantly DN or CD4^–^CD8^+^ iNKT cells. There was no significant recovery in peripheral blood CD4^+^ iNKT cells in the first 4 weeks of ART, despite increased peripheral blood CD4 count [25].

Our findings are consistent with prior human studies measuring iNKT cells in HIV infection, which report reduced iNKT cell frequency in PLWH [23, 30, 31]. A previous study demonstrated that in vitro HIV-1 infection directly infects and selectively depletes CD4^+^ iNKT cells. Activated iNKT cells were more susceptible to HIV-1 infection than conventional CD4 T cells [32]. In HIV/leprosy coinfection, iNKT cell populations were found to be reduced more profoundly than in leprosy or HIV infection alone [33]. iNKT cell activation due to mycobacterial infection might exacerbate iNKT cell depletion in PLWH. Although we found the lowest iNKT cell frequency and numbers in patients with HIV-1 infection and active TB, active TB did not clearly have an additive effect of iNKT cell depletion in PLWH.

There are a number of potential mechanisms by which iNKT cells may contribute to immunopathology in TB-IRIS. They may directly recognize foreign or self-lipid antigens presented via CD1d, or become activated by local cytokine networks [26]. Once activated, iNKT cells may rapidly secrete proinflammatory cytokines and chemokines promoting CD4 T-cell expansion, activation, and neutrophil infiltration (features of TB-IRIS we have previously shown), in addition to causing cell death [5, 6, 9, 10, 26, 34]. Ultimately, this cascade may lead to matrix metalloproteinase activation and tissue destruction, in turn propagating proinflammatory cytokine secretion in the vicious cycle of hyperinflammation that is the hallmark of TB-IRIS [7, 25].

iNKT cell quantification using α-galcer–loaded CD1d-loaded tetramers is recognized as a stringent method of iNKT cell quantification [35, 36]. However, we cannot extrapolate our findings beyond the limitations of this methodology, which may be affected by T-cell receptor downregulation on activation, nor beyond peripheral blood into tissue compartments [35]. It is possible that increased circulating iNKT cells in TB-IRIS patients represent failure of migration to tissues. We found evidence of increased iNKT cell degranulation in extrapulmonary TB, compared to pulmonary TB, raising the possibility that more abundant, disseminated *M. tuberculosis* antigen may drive iNKT cell degranulation in HIV-associated TB and increased iNKT cell cytotoxicity in TB-IRIS patients [24].

As a rare T-cell subset, iNKT cells have formerly been difficult to study and iNKT cell function in infection is a relatively understudied area. However, in the field of oncology, adjuvants to boost iNKT cell cytotoxicity have been the focus of translational research and have entered early-phase clinical trials [37, 38]. Our study suggests that boosting iNKT cell cytotoxicity would not be an appropriate strategy in HIV-associated TB. However, an improved understanding of the role of iNKT cells in TB immunopathology could identify novel therapeutic targets. As human and mouse iNKT cell physiology differ, further human clinical and cellular studies are required, including study of iNKT cells in tissue compartments.

## CONCLUSIONS

This study supports a role for iNKT cells in innate immune dysfunction in paradoxical TB-IRIS. We have shown profound CD4^+^ iNKT cell subset depletion in advanced HIV-1 infection and a lesser effect of active TB in HIV-uninfected patients. In patients with advanced HIV and a new diagnosis of active TB, iNKT cell populations were skewed towards a proinflammatory, cytotoxic phenotype. Patients who developed TB-IRIS had increased iNKT cells compared to those without IRIS and iNKT cell degranulation occurred at the time of IRIS, potentially contributing to immunopathology.

## Supplementary Data

Supplementary materials are available at *Clinical Infectious Diseases* online. Consisting of data provided by the authors to benefit the reader, the posted materials are not copyedited and are the sole responsibility of the authors, so questions or comments should be addressed to the corresponding author.

ciz501_Suppl_Supplementary_Material

## Notes

***Author contributions.*** N. F. W., G. M., R. J. W., J. S. F., P. T. E., and K. A. W. conceived and designed the clinical study. N. F. W., G. M., and R. J. W. recruited the clinical cohort. N. F. W., N. J., R. J. W., and K. A. W. conceived and designed the cellular studies. N. F. W. and K. A. W. conducted the cellular studies. N. F. W. and C. O. performed data analysis. N. F. W. and K. A. W. hold all primary data and are responsible for the integrity of the data. All authors contributed to the writing of the manuscript and approved the final submitted version.

***Acknowledgments.*** The authors thank S. Mansour for review of the draft manuscript.

***Financial support.*** N. F. W. received funding from Wellcome (grant number 094000), the British Federation of Women Graduates (Elizabeth Bowden scholarship), and the Federation of African Immunological Societies and is currently supported by a National Institute for Health Research Academic Clinical Lecturership, the British Infection Association (Research Project Grant), and a Starter Grant for Clinical Lecturers (Academy of Medical Sciences UK, Wellcome, Medical Research Council UK, British Heart Foundation, Arthritis Research UK, Royal College of Physicians, and Diabetes UK). G. M. was supported by Wellcome (grant numbers 098316 and 203135/Z/16/Z), the South African Research Chairs Initiative of the Department of Science and Technology, the National Research Foundation (NRF) of South Africa (grant number 64787), NRF incentive funding (UID: 85858), and the South African Medical Research Council through its Tuberculosis and Human Immunodeficiency Virus Collaborating Centres Programme with funds received from the National Department of Health (RFA number SAMRC-RFA-CC: TB/HIV/AIDS-01-2014). R. J. W. and K. A. W. receive support from the Francis Crick Institute, which is supported by Wellcome (grant number FC001218), Medical Research Council (grant number FC001218), Cancer Research UK (grant number FC001218), and the European Union Horizon 2020 research and innovation program (grant agreement number 643381). R. J. W. receives additional support from Wellcome (grant numbers 104803 and 203135), the National Institutes of Health (grant number U01AI115940), and the European and Developing Countries Clinical Trials Partnership. N. J. was supported by Wellcome (grant number 088316) and the South African NRF (grant number 443386).

***Potential conflicts of interest.*** The authors: No reported conflicts of interest. All authors have submitted the ICMJE Form for Disclosure of Potential Conflicts of Interest. Conflicts that the editors consider relevant to the content of the manuscript have been disclosed.
