# Supplementary material for: Invariant Natural Killer T-cell Dynamics in Human Immunodeficiency Virus–associated Tuberculosis
Source: Clin Infect Dis. 2019 Jun 12;70(9):1865–74. doi: 10.1093/cid/ciz501 (PMC7156773; doi:10.1093/cid/ciz501)
Supplement: ciz501_Suppl_Supplementary_Material [file ciz501_suppl_supplementary_material.docx]

Online Data Supplement

**Invariant Natural Killer T cell dynamics in HIV-associated tuberculosis**

*Invariant Natural Killer T cells in TB-IRIS*

Walker NF, Opondo C, Meintjes G, Jhilmeet N, Friedland JS,

Elkington PT, Wilkinson RJ, Wilkinson KA.

**Materials and Methods**

*Study Participants and clinical assessment*

The study was approved by the University of Cape Town Human Research Ethics Committee (REF 516/2011) and conducted in accordance with the Declaration of Helsinki. Cross-sectional study participants were recruited in an outpatient clinic in Khayelitsha, South Africa and were either healthy volunteers, patients with symptoms requiring assessment, or recently diagnosed TB patients. HIV-infected patients were ART naive at enrolment. Cross-sectional study participants who were on anti-tuberculosis therapy were required to have had less than 3 doses prior to study samples being collected. Once enrolled, cross-sectional study participants were provided appropriate follow up by the research team for study clinical results and were then followed up routinely by clinic staff, unless also eligible for the longitudinal study.

Cross-sectional study participants were retrospectively designated into four categories:

1) HIV-uninfected patients without active TB (HIV-TB-)

2) HIV-uninfected patients with a new diagnosis of active TB (HIV-TB+)

3) ART naïve, HIV-infected patients without active TB (HIV+TB-)

4) ART naïve, HIV-infected patients with a new diagnosis of active TB (HIV+TB+).

Active TB was diagnosed on the basis of smear or culture positivity, or in cases of smear-negative TB according to international guidelines [1, 2]. This required suggestive symptoms and at least one of:

1. Sputum smear positive for acid fast bacilli on microscopy (Smear positive)
2. Sputum Gene Xpert-RIF (Cepheid, Sunnyvale, CA) positive for *Mycobacterium tuberculosis* (Mtb)
3. Sputum culture positive for Mtb (Culture-confirmed)
4. Clinical features highly suggestive of TB such as diagnostic features on chest radiograph or other imaging modality and a decision to start TB treatment by the treating clinician (Clinical diagnosis)

Cross-sectional study participants who had symptoms but did not meet the criteria for TB diagnosis were designated controls if there was a low clinical suspicion for active TB and they had at least one induced sputum smear negative for acid fast bacilli and one induced sputum culture that was negative for Mtb. These patients were not started on anti-tuberculosis therapy. All HIV-infected patients who were designated non-TB controls also had at least one induced sputum smear negative for acid fast bacilli and one induced sputum culture that was negative for Mtb*.* HIV-infected active TB patients in the cross-sectional study who met eligibility criteria for the longitudinal study were co-enrolled, or if eligibility became apparent after enrolment, were invited to subsequently participate in the longitudinal study.

Longitudinal study participants were ART naïve HIV-infected patients with a low CD4 count (<200 cells/mm^3^) and a recent diagnosis of active TB. Following anti-tuberculosis therapy initiation and enrolment into the longitudinal study (study visit TB0) patients received counselling for ART initiation. Anti-tuberculosis therapy and ART followed national guidelines [3, 4]. First-line ART at the time of the study was principally tenofovir, lamivudine, and efavirenz. ART was initiated typically two weeks after anti-tuberculosis therapy (study visit ARV0) and patients attended further scheduled study visits at two weeks post-ART initiation (ARV2, Day 14 +/- 72 hours) and four weeks post-ART initiation (ARV4, Day 28 +/-72 hours) for clinical assessment and sampling. Patients who had taken more than four doses of TB treatment at enrolment contributed samples from ARV0.

Patients were requested to attend for assessment if any new symptoms or clinical deterioration occurred (study interim assessment) and were followed up to twelve weeks post-ART initiation. Clinical research staff telephoned participants regularly to reinforce this, to remind patients about scheduled visits and to investigate non-attendance. If a case of TB-IRIS was suspected, study samples were collected as at a scheduled visit, in addition to clinically indicated diagnostic tests. Where possible, when patients were hospitalized at the time of a study visit, they were visited by the study team for data and sample collection. Retrospective designation into one of three longitudinal study categories (paradoxical TB-IRIS (INSHI IRIS), probable paradoxical TB-IRIS not meeting INSHI criteria (IRIS non-INSHI), and no paradoxical TB-IRIS (non-IRIS)) followed the results of all relevant investigations and clinical follow up, and was made on case review by a consensus panel (comprising the study clinician NFW, and two clinical specialists: GM, RJW). Designation as TB-IRIS included both INSHI-IRIS and non-INSHI IRIS.

In both cross-sectional and longitudinal studies, demographic information was recorded at enrolment, including gender and smoking status. At each study visit, symptoms and clinical examination, full blood count, albumin, C-reactive protein (CRP) and chest radiograph were performed, plus additional investigations if clinically indicated. Induced sputum and venous blood were collected (see below) at each visit for microbiological and laboratory analysis.

Venous blood for PBMC isolation was collected in sodium heparin vacutainers, transported at room temperature to UCT, and processed within four hours of collection. PBMC were isolated by layering over Ficoll and cryopreserved in heat-inactivated fetal calf serum (FCS) with 10% dimethyl sulfoxide (DMSO) until used in batches.

Additional venous blood sample analysis was performed by the National Health Laboratory Service (NHLS), including full blood count and differential measured using a Siemens Advia 2120I (Siemens, Surrey, UK), albumin and C-reactive protein (CRP) quantification on a Roche Modular (F. Hoffman-La Roche Ltd) and for HIV-infected patients, CD4 count measured on a Beckman Coulter FC500MP (Beckmann Coulter, Inc, Buckinghamshire, UK) and HIV-1 viral load measured on an Abbott M2000 (Abbott Analytical Limited, London, UK). Induced sputum was collected for microscopy and mycobacterial culture.

*CD1d Tetramer staining*

Cryopreserved PBMC were rapidly thawed in warmed RPMI/10% FCS and washed. All wash steps consisted of centrifugation at 1500 rpm for 5 minutes, discarding of supernatant and disruption of the cell pellet by vortex. PBMC were counted using a Bio-rad TC20^TM^ automated cell counter and viability was ascertained by trypan blue exclusion. For iNKT cell enumeration, one million cells per tube were transferred into two labelled fluorescence activated cell sorting (FACS) tubes per patient: one for α-galcer-loaded CD1d tetramer and one for CD1d (control) tetramer staining for each sample. Cells were washed in PBS and resuspended for viability staining with Violet LIVE/DEAD**^©^** Fixable stain kit (VIVID, Invitrogen, Paisley, UK) (1:1000 dilution, 200μl per tube) and incubated for 30 minutes at 4^o^C in the dark. They were then washed in PBS and resuspended in 30μl cold PBS prior to tetramer staining with α-galcer-loaded CD1d tetramer (tet+) or control CD1d tetramer (tet-cont) (Proimmune, Oxford, UK), added at 0.5ul per tube and then incubated for 30 minutes on ice. Tet+ and tet-cont-stained cells were protected from light at all times.

*Characterisation by cell surface marker expression*

Following tetramer staining, cells for surface marker characterisation were stained with an antibody mastermix containing CD3, CD19, CD4, CD8, CD107a, CD95, CD161, CD40L, PD1, and Vβ11 for 30 minutes at 4^o^C (see Supplementary Table S1). They were then washed and re-suspended in PBS, 1% Hi-FCS, 2% paraformaldehyde for 1 hour. After a further wash, cells were re-suspended in 300μl wash buffer and acquired on an LSR Fortessa (BD Biosciences) within 24 hours of staining. Single fluorochrome-stained positive and negative control compensation beads were acquired for each experiment to enable fluorescence compensation. Data were analysed using Flowjo software (Tree Star, Ashland, OR).

iNKT cell frequency was calculated as a percentage of CD3+ CD19- live lymphocytes, with subtraction of the equivalent tet-cont proportion, and reported per million CD3+CD19- live lymphocytes. Negative values were reassigned zero for analysis. Zero values were assigned 1 if graphically represented on a log scale. Absolute iNKT cell numbers were calculated by multiplying the iNKT cell count, as a percentage of live lymphocytes, with the total lymphocyte count per milliliter of peripheral blood, as previously reported [5]. Phenotypic characteristics of iNKT cells are reported as the iNKT cell percentage expressing cell surface markers (CD4, CD8, CD107a, CD95, CD161, CD40L, PD1). This proportion was multiplied by the total iNKT cell frequency to give the iNKT cell subset frequency.

*Statistical analysis*

Flow cytometry data was analysed using Flowjo software (Treestar, USA). Gating was determined by comparison to samples stained by the control tetramer or fluorescence-minus-one controls. Statistical analysis was performed using Prism 6 (GraphPad, UK) and STATA version 14. Unadjusted non-parametric analyses were by two-tailed Fisher’s Exact or Mann–Whitney U, or for comparisons of more than two groups, by Kruskal-Wallis with Dunn’s multiple comparisons test. Comparison of total CD4 count changes over time in the longitudinal study was by two-way repeated measures ANOVA. In the cross-sectional study, we used a multivariable linear regression model to investigate differences in  iNKT cell frequency and percentage CD4/CD8 expression by disease category. In the longitudinal study, a multivariable negative binomial model was fitted to examine the association of iNKT frequency with TB-IRIS status and a multivariate linear regression model was used to estimate the difference in CD4/CD8 cell subset percentages between TB IRIS and non-IRIS patients. Data from scheduled visits, ARV0, ARV2 and ARV4 was included in this analysis and ARV0 was used as the baseline timepoint.

**Supplementary References**

1. Siddiqi K, Lambert ML, Walley J. Clinical diagnosis of smear-negative pulmonary tuberculosis in low-income countries: the current evidence. Lancet Infect Dis **2003**; 3(5): 288-96.

2. WHO. Improving the diagnosis and treatment of smear-negative pulmonary and extra-pulmonary tuberculosis among adults and adolescents: recommendations for HIV-prevalent and resource-constrained settings. World Health Organisation, Geneva, **2006**.

3. The South African Antiretroviral Treatment Guidelines 2013. Department of Health, Republic of South Africa, **2013**.

4. National Tuberculosis Management Guidelines 2014: Department of Health, Republic of South Africa 2014.

5. Kee SJ, Kwon YS, Park YW, et al. Dysfunction of Natural Killer T Cells in Patients with Active *Mycobacterium tuberculosis* Infection. Infect Immun **2012**; 80(6): 2100-8.

**Supplementary Figure Legends**

**Supplementary Figure S1 iNKT cell enumeration in cross-sectional study participants**

iNKT cells were enumerated using α-galcer-loaded CD1d tetramers and control CD1d tetramers (no α-galcer). Gating on CD3+ CD19- live lymphocytes generated the plots shown. For each participant, peripheral blood mononuclear cells were stained using α-galcer-loaded CD1d tetramers (column A) in parallel with a control tetramer (column B). iNKT cells (shown in Q2, column A) were defined as CD3+ CD19- CD1d α-galcer tet+ Vβ11+ T cells and enumerated by subtraction of non-specific tetramer staining in the equivalent control gate (Q2 column B). Representative plots are shown for each of the four patient categories in the cross-sectional study: HIV-uninfected patients without active TB (HIV-TB-); HIV-uninfected patients with active TB (HIV-TB+); HIV-1-infected patients without active TB (HIV+TB-); HIV-1-infected patients with active TB (HIV+TB+).

**Supplementary Figure S2 CD4 and CD8 iNKT cell subset frequency in cross-sectional study patients**

CD4+CD8- iNKT cell subset frequency (cells per million CD3+CD19- live lymphocytes) was reduced in HIV infection and in active TB in HIV-uninfected patients (A). There was reduced CD4-CD8- iNKT cell frequency in HIV-infected patients with active TB compared to HIV-uninfected patients without active TB (B). There was no difference in iNKT cell frequency of CD-CD8+ (C) or CD4+ CD8+ (D) iNKT cell frequency between HIV-infected and uninfected patients, with and without active TB. Analysis was by Kruskal Wallis with Dunn’s multiple comparison’s test to calculate multiplicity adjusted p values: *p<0.05; **p<0.01; ***p<0.001.

**Supplementary Figure S3 iNKT cell numbers in longitudinal study patients**

iNKT cells were enumerated by flow cytometry using ⍺-galcer loaded CD1d tetramers, in a cohort of 46 HIV-1-infected patients with active TB. Following TB diagnosis (TB0), TB treatment was initiated. Anti-retroviral therapy was initiated at ARV0, a median of 17.5 days post TB treatment initiation. Patients who had taken more than four doses of TB treatment at enrolment contributed data from ARV0, resulting in fewer data points at TB0. Additional study visits occurred at two (ARV2) and four (ARV4) weeks post-ART initiation. TB-IRIS presentation was typically at ARV2. iNKT cell numbers were calculated by multiplying the iNKT cell count, as a percentage of live lymphocytes quantified by flow cytometry, with the total lymphocyte count per millilitre of peripheral blood as recorded on the full blood count. There was a trend towards increased iNKT cell numbers in TB-IRIS patients compared to non-IRIS controls in the age and sex-adjusted multivariate model (p=0.062). Zero values were replaced by one for representation on a log scale.

**Supplementary Figure S4 CD4 negative iNKT cell subsets predominate in TB-IRIS and non-IRIS patients**

CD4-CD8- and CD4-CD8+ iNKT cell subset percentage (A, B) and iNKT cell frequency per million CD3+ CD19- live lymphocytes (C, D) are shown. CD4-CD8- iNKT cells were the most abdundant as a percentage of total iNKT cells (A), followed by CD4-CD8+ iNKT cells (B) in both TB-IRIS and non-IRIS patients. However, in TB-IRIS patients CD4-CD8- (C) and CD4-CD8+ (D) iNKT cell frequency was increased compared to non-IRIS patients at ARV2, the usual time of IRIS presentation. CD4+CD8+ iNKT cells were infrequent (data not shown). Zero values were replaced by 0.1 for representation on a log scale. Analysis was by Mann Whitney-U comparing TB-IRIS with non-IRIS at each timepoint: *p<0.05.

**Supplementary Figure S5 iNKT cell phenotype in HIV-associated TB**

iNKT cells where characterized by proportional surface expression of CD161, CD107a, CD95, PD1 and CD40L, indicating maturation, degranulation, cytotoxicity, anergy and activation, respectively. CD161+ (A) and CD107a+ (B) iNKT cell proportions were dynamic. CD95+ iNKT cell proportions were very high in both TB-IRIS and non-IRIS patients (C). PD1+ iNKT cell proportions were relatively high (E) whereas CD40L+ iNKT cell proportions were relatively low, possibly indicating an exhausted state (F). Individual patient data are shown and consecutive visits are joined by a line. Routine study visits were at ARV0 (anti-retroviral therapy initiation), ARV2 and ARV4 (two and four weeks post-ART initiation). Unscheduled study visits indicating new symptoms were at ARV1 and ARV3 (one and three weeks post-ART initiation).

**Supplementary Tables**

**Supplementary Table S1** Flow cytometry panel for iNKT cell enumeration and phenotype, comprising LIVE/DEAD**^©^** Fixable stain (VIVID), either α-galcer-loaded CD1d tetramer or control CD1d tetramer and antibodies for cell surface markers (surface antibody mastermix 1).

**Supplementary Table S2** Multivariable linear regression model of CD4/CD8 percentage expression on iNKT cells in the cross-sectional cohort by clinical category in comparison to HIV-uninfected controls without active TB (HIV-TB-), adjusted for age and sex.

|  |  | Coefficient | 95% CI | p value |
| --- | --- | --- | --- | --- |
| CD4-CD8+ |  |  |  |  |
|  | HIV-TB+ | 4.92 | -3.58, 13.4 | 0.257 |
|  | HIV+TB- | 6.97 | 0.42, 13.5 | **0.037** |
|  | HIV+TB+ | 12.4 | 2.28, 22.5 | **0.016** |
| CD4+CD8+ |  |  |  |  |
|  | HIV-TB+ | -0.38 | -2.49, 1.73 | 0.725 |
|  | HIV+TB- | -0.39 | -1.18, 0.40 | 0.334 |
|  | HIV+TB+ | 2.22 | -1.87, 6.31 | 0.288 |
| CD4+CD8- |  |  |  |  |
|  | HIV-TB+ | -4.00 | -17.9, 9.91 | 0.573 |
|  | HIV+TB- | -23.8 | -33.7, -13.8 | **<0.001** |
|  | HIV+TB+ | -30.5 | -41.3, -19.6 | **<0.001** |
| CD4-CD8- |  |  |  |  |
|  | HIV-TB+ | 0.45 | -13.2, 14.1 | 0.949 |
|  | HIV+TB- | 13.6 | 3.31, 23.8 | **0.010** |
|  | HIV+TB+ | 11.8 | -4.45, 28.0 | 0.155 |

**Supplementary Table S3** Multivariable negative binomial model assessing relationship between iNKT cell frequency and TB-IRIS status.

| Unadjusted | Coefficient | 95% CI | p value |
| --- | --- | --- | --- |
|  | 3.99 | 1.18, 13.5 | **0.026** |
| Adjusted^a^ | Coefficient | 95% CI | p value |
|  | 4.13 | 1.23, 13.9 | **0.022** |

^a^adjusted for age and sex

**Supplementary Table S4** Multivariate linear regression model to estimate the difference in CD4/CD8 cell subset percentage between TB IRIS and non-IRIS patients, adjusted for time.

|  | Difference in cell subset percentage (TB-IRIS vs non-IRIS) | 95% CI | p value |
| --- | --- | --- | --- |
| CD4-CD8+ | -3.02 | -14.4, 8.36 | 0.603 |
| CD4+CD8+ | 1.03 | -10.5, 12.6 | 0.861 |
| CD4+CD8- | -6.64 | -12.0, -1.28 | **0.015** |
| CD4-CD8- | 8.63 | -5.49, 22.2 | 0.231 |

**Supplementary Figures**

**Supplementary Figure S1**

**Supplementary Figure S2**

**Supplementary Figure S3**

**Supplementary Figure S4**

**Supplementary Figure S5**
